# The mechanism and clinical application of DNA damage repair inhibitors combined with immune checkpoint inhibitors in the treatment of urologic cancer

**DOI:** 10.3389/fcell.2023.1200466

**Published:** 2023-05-25

**Authors:** Deqian Xie, Bowen Jiang, Shijin Wang, Qifei Wang, Guangzhen Wu

**Affiliations:** Department of Urology, The First Affiliated Hospital of Dalian Medical University, Dalian, China

**Keywords:** urologic cancer, DNA damage repair, immune checkpoint inhibitors, immunotherapy, combination therapy, tumor microenvironment

## Abstract

Urologic cancers such as kidney, bladder, prostate, and uroepithelial cancers have recently become a considerable global health burden, and the response to immunotherapy is limited due to immune escape and immune resistance. Therefore, it is crucial to find appropriate and effective combination therapies to improve the sensitivity of patients to immunotherapy. DNA damage repair inhibitors can enhance the immunogenicity of tumor cells by increasing tumor mutational burden and neoantigen expression, activating immune-related signaling pathways, regulating PD-L1 expression, and reversing the immunosuppressive tumor microenvironment to activate the immune system and enhance the efficacy of immunotherapy. Based on promising experimental results from preclinical studies, many clinical trials combining DNA damage repair inhibitors (e.g., PARP inhibitors and ATR inhibitors) with immune checkpoint inhibitors (e.g., PD-1/PD-L1 inhibitors) are underway in patients with urologic cancers. Results from several clinical trials have shown that the combination of DNA damage repair inhibitors with immune checkpoint inhibitors can improve objective rates, progression-free survival, and overall survival (OS) in patients with urologic tumors, especially in patients with defective DNA damage repair genes or a high mutational load. In this review, we present the results of preclinical and clinical trials of different DNA damage repair inhibitors in combination with immune checkpoint inhibitors in urologic cancers and summarize the potential mechanism of action of the combination therapy. Finally, we also discuss the challenges of dose toxicity, biomarker selection, drug tolerance, drug interactions in the treatment of urologic tumors with this combination therapy and look into the future direction of this combination therapy.

## 1 Introduction

Urological cancers (UC) are malignant tumors originating from parts related to the urinary system, such as the kidney, ureter, bladder, prostate, and urethra. They are among the most common types of malignant tumors worldwide (Rouprêt et al., 2011). Among them, kidney, bladder, and prostate cancer are the three most predominant types of UC, and the prevalence and mortality of these three types of UC are increasing (Miyazaki and Nishiyama, 2017; Montironi and Cimadamore, 2022; Netto et al., 2022). Traditional treatments such as surgical resection, chemotherapy, and radiotherapy are generally used in clinical practice for UC. Although these methods can limit the progression of UC to a certain extent, the efficacy of these therapies is extremely limited for advanced and recurrent tumors, and patients have a low survival rate and poor prognosis (Kong et al., 2022). Also, the extensive cell-killing mechanism of radiotherapy can cause serious adverse effects on patients and affect their survival and quality of life (Ji et al., 2022).

UC immunotherapy works by activating the patient’s immune system to combat tumor progression. Compared with traditional treatments, immunotherapy has more precise selectivity, is less damaging to healthy cells, and has a lower probability of adverse effects (Vasekar et al., 2016). In addition, immunotherapy can reverse the immunosuppressive tumor microenvironment (TME) which tumor cells form by releasing immunosuppressive factors and reducing immunogenicity, regulate the number and activity of immune cells in the TME, and promote the body’s immune system to attack UC cells (Yang, 2015; Saleh and Elkord, 2020). Clinical immunotherapy for UC usually uses immune checkpoint blockade (ICB) and chimeric antigen receptor T-cell (CAR-T cell) immunotherapy (Kennedy and Salama, 2020). ICB uses immune checkpoint inhibitors (ICIs) to inhibit the action of immune checkpoints such as PD-1, PD-L1, and CTLA-4, thereby enhancing the susceptibility of tumor cells to attack by the immune system (Naimi et al., 2022; Yamaguchi et al., 2022). Among the therapies for bladder cancer, the FDA has approved five ICIs, including the PD-L1 immune checkpoint inhibitors atezolizumab and durvalumab, to treat locally advanced and metastatic bladder cancer (Rouanne et al., 2018). In addition, the FDA also approved the PD-1/PD-L1 immune checkpoint inhibitors nivolumab and pembrolizumab for treating metastatic kidney cancer (Chan et al., 2022). However, the limitations of immunotherapy for UC cannot be ignored. On the one hand, tumor cells can evade the immune system by reducing the expression of tumor antigens and activating immunosuppressive pathways. On the other hand, as tumor cells may mutate during immunotherapy, this further reduces the ability of the patient’s immune system to attack tumor cells, reducing the efficacy of immunotherapy (Walsh et al., 2019; Bagchi et al., 2021).

To address the limitations of immunotherapy and improve its efficacy, researchers are developing various combination therapies that address multiple pathways of cancer progression and immune evasion. Among them, the combination of DNA damage repair (DDR) inhibitors and ICIs is one of the most promising therapies in development (Shi et al., 2022). DDR is a complex biological process that repairs DNA damage caused by endogenous or exogenous factors, ensuring the integrity of genomic DNA to maintain normal cellular function and genetic stability (Huang and Zhou, 2021). However, DDR is two-sided, as tumor cells can use DDR to resist immunotherapeutic attacks, leading to the failure of immunotherapy (Hopkins et al., 2022). Therefore, applying DDR inhibitors can interfere not only with the immune system but also with the DDR process in some tumor cells, leading to DDR failure and increased tumor cell death. In addition, applying DDR inhibitors to some tumor cells can promote the release of tumor antigens, activate immune pathways, and promote the release of inflammatory cytokines to reverse the immunosuppressive TME, sensitize tumor cells to immunotherapy, and improve the efficacy of immunotherapy (Pilié et al., 2019; Catalano et al., 2022).

In this review, we describe the concept and pathway of DDR. We highlight the results of preclinical and clinical trials of different DDR inhibitors combined with ICIs in UC and summarize the possible mechanisms of action of the combination therapy in immunotherapy for UC. Finally, we also explore the challenges faced by therapies combining DDR inhibitors with ICI in UC treatment and provide an outlook on the future of this combination therapy. This review may provide a theoretical basis and practical guidance for the future immunotherapy of patients with UC and instill new hope in them.

## 2 DNA damage repair

### 2.1 DNA damage

**FIGURE 1:**
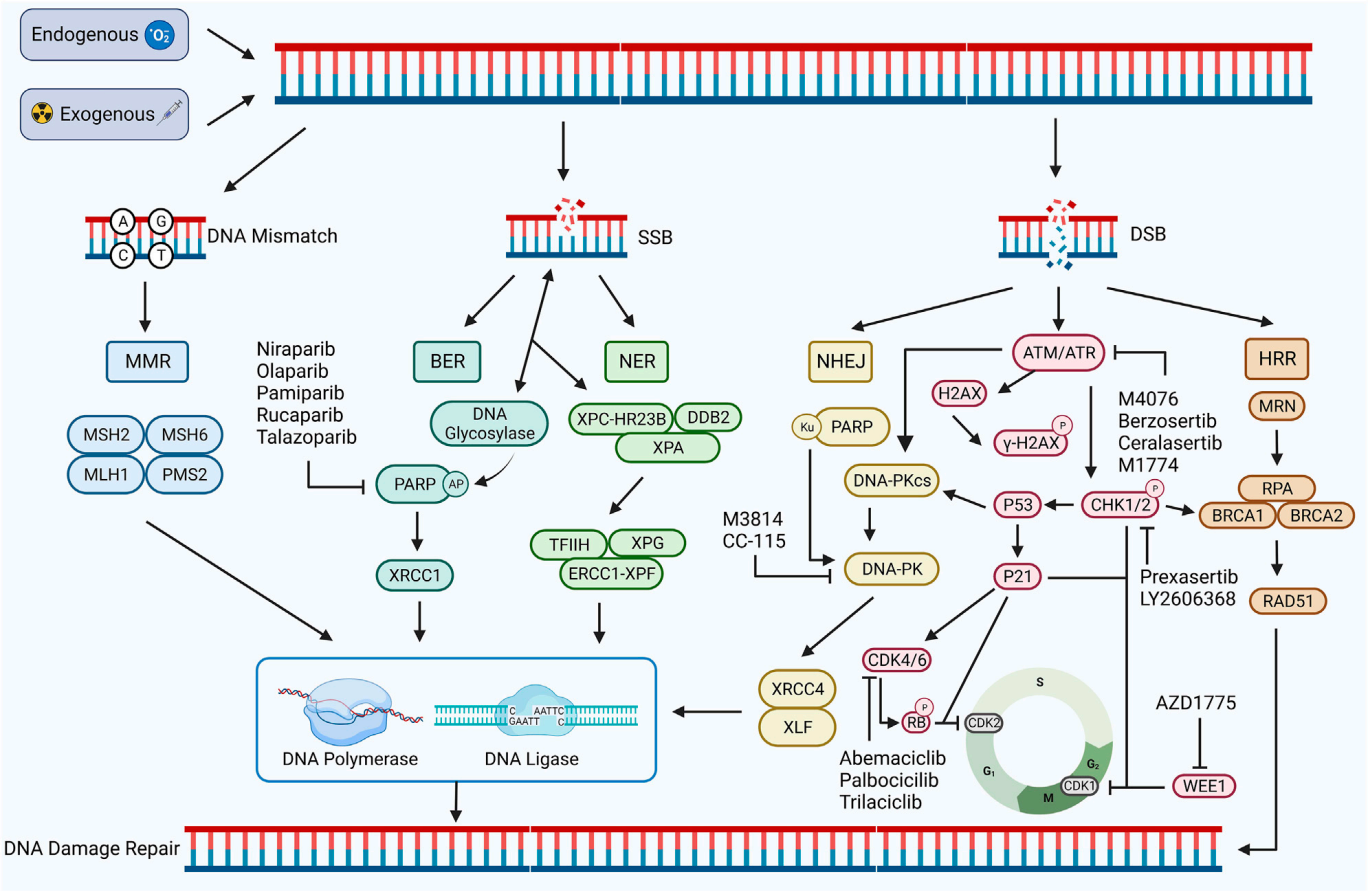
DDR damage repair pathway. DNA damage occurs in response to exogenous or endogenous factors, and the DDR pathway is activated. Mismatched DNA is repaired through the MMR pathway, and SSB is repaired through the BER and NER pathways. Both HRR and NHEJ pathways repair DSB. HRR is a precise repair of damaged DNA strands using undamaged sister chromatids as templates, while NHEJ is a highly error-prone DDR pathway. PARP, ATM, ATR, CHK1/2, CDK4/6, WEE1, DNA-PK, and other proteins play important roles, including regulating the cell cycle and promoting the damage repair process. Inhibition of the expression of these important proteins can inhibit the process of DDR and contribute to the inability of damaged DNA to complete repair. Created with BioRender.com.

DNA damage refers to a disruption or alteration of the chemical structure of a DNA molecule that results in the impairment or complete loss of the function of the DNA(22). DNA damage can be induced by both endogenous and exogenous factors. Endogenous factors include DNA replication, errors in repair processes, and reactive oxygen radicals generated during oxidative stress, whereas exogenous factors include chemical agents, physical factors, and viral infections, all of which can directly or indirectly affect the DNA molecule (Tubbs and Nussenzweig, 2017; Carusillo and Mussolino, 2020). DNA damage can be divided into two types: single-stranded breaks (SSBs) and double-stranded breaks (DSBs). SSB generally refers to the occurrence of base damage on the DNA strand and single-strand breaks, which are relatively easy to repair. DSB generally refers to a situation where both DNA strands are broken, cross-linked, or missing simultaneously (Carusillo and Mussolino, 2020). SSBs can be converted to DSBs if not repaired in time, which is extremely complex and threatening to the organism (Khoronenkova and Dianov, 2015) (Figure 1).

### 2.2 DDR pathway

DDR, in turn, refers to the maintenance of DNA stability and the integrity of genetic information by specific molecules and pathways that enable cells to restore damaged DNA to its normal structure and function (Karzai et al., 2018). DDR pathways are of five types: mismatch repair (MMR), base excision repair (BER), nucleotide excision repair (NER), homologous recombination repair (HRR), and non-homologous end joining repair (NHEJ) (Li et al., 2020). Among these, MMR is used to repair DNA replication errors and mismatches, BER and NER to repair SSBs, and HRR and NHEJ are important repair pathways for DSBs (Bhattacharjee and Nandi, 2016; Peyraud and Italiano, 2020) (Figure 1).

#### 2.2.1 Mismatch repair (MMR)

MMR occurs mainly during the cell cycle’s S phase (Schroering et al., 2007). During MMR, the MSH family MSH2:MSH6 or MSH2:MSH3 complexes recognize and bind to the mismatch site on the DNA strand. PMS2 and MLH1 of the MLH family form a nuclease complex with the MSH complex, and this complex can cut the strand containing the mismatch site, produce a single-strand break, and remove the DNA strand containing the mismatch site through the combined action of nucleic acid exonucleases, then complete the DNA strand repair by the action of DNA polymerase and ligase (Fishel, 2015; He et al., 2022a). An abnormal MMR process results in DNA damage that cannot be repaired in time, resulting in microsatellite instability (MSI). MSI leads to several mutations in tumor cells, which increase the tumor mutational burden (TMB) and enhance tumor immunogenicity, activating the body’s anti-tumor immune response, which inhibits tumor development by recognizing and killing tumor cells. Also, MSI status promotes the expression of PD-L1, which is closely related to ICB (Salem et al., 2020; He et al., 2022a).

#### 2.2.2 Base excision repair (BER)

BER occurs mainly during the cell cycle’s G1 and S phases. DNA glycosylase can recognize and shear the damaged bases during BER to form base-free nucleotides (AP). Poly ADP-ribose polymerase (PARP) can recognize and bind to AP and recruit AP nucleic acid endonucleases to cleave AP to form a single-strand break. Finally, the interaction of XRCC1, DNA ligase, and DNA polymerase completes the repair of SSB (Demin et al., 2021; Hirota et al., 2022). Defects in the BER pathway may lead to the accumulation of DNA damage and promote the formation of DSBs. It can also inhibit tumor development, enhance tumor sensitivity to treatment, and lead to massive tumor cell death (Srivastava and Raghavan, 2015).

#### 2.2.3 Nucleotide excision repair (NER)

NER occurs mainly during the cell cycle’s G1 and S phases, where XPC-HRR23B and DDB2 recognize and bind the damaged DNA and recruit XPA proteins to form complexes. The damaged DNA strand is excised by transcription factors TFIIH, XPG, and ERCC1-XPF, and DNA polymerase and DNA ligase complete the SSB repair (Saijo et al., 2011). Defects or inhibition of ERCC1-XPF can affect the function of the NER, reducing the ability of tumor cells to effectively repair cisplatin-induced DNA damage and thus increasing tumor cell mortality (Kirschner and Melton, 2010; Tang et al., 2011).

#### 2.2.4 Homologous recombination repair (HRR)

HRR is a pathway for precisely repairing DNA double-strand breaks using undamaged sister chromatids as templates, mainly during the S phase of the cell cycle with chromosome replication and during the G2 phase (Arnoult et al., 2017). During HRR, the MRN complex consisting of MRE11, RAD50, and NBS1 recognizes and binds DNA ends facilitated by CtIP nucleases to generate single-stranded DNA (ssDNA). ssDNA is encapsulated by replication protein A (RPA) to avoid degradation or DNA secondary structure formation (Langerak et al., 2011; Shi et al., 2021). Meanwhile, DSB activates the protein kinases ATR and ATM, activates the downstream signal CHK1, phosphorylates BRCA1/BRCA2, and promotes BRCA1/BRCA2-mediated replacement of RPA by RAD51, forming RAD51-ssDNA nucleoprotein filaments. This further mediates the search for homologous sequences on sister chromatids and the formation of D-loop triple helix structures, completing the repair of the DSB (Jazayeri et al., 2006; Foo et al., 2021). In addition, ATM and ATR can phosphorylate CHK1 and CHK2, inhibiting CDK activity and leading to cell arrest in the G2 phase, providing sufficient time and conditions for HRR repair (Smith et al., 2010). WEE1 is not affected by DDR but can also inhibit CDK kinase activity, prolong the G2 phase, prevent premature mitosis in cells with damaged DNA, maintain DNA stability in concert with the HRR pathway, and avoid cell death (Elbæk et al., 2020). Defects in the BER process lead to the accumulation of SSBs and the formation of DSBs because DNA polymerase stops replication when it encounters SSBs. This creates single-stranded regions on the other strand that are susceptible to degradation by nucleases or attack by other damage factors, forming DSBs (Khoronenkova and Dianov, 2015). Defects in the HR pathway in the G1 and G0 phases of the cell cycle lead to the inability to repair DSBs, which in turn leads to synthetic lethal effects, contributing to the death of more tumor cells (Setton et al., 2021). In addition, defective BRCA1/BRCA2 in tumor cells may also lead to failure of DSB repair by the HRR pathway, resulting in massive tumor cell death (Wanderley et al., 2022).

#### 2.2.5 Non-homologous end-joining repair (NHEJ)

NHEJ is an error-prone double-stranded DDR pathway, which, unlike the HRR pathway, can occur at all cell cycle stages because it does not require homologous DNA as a template (Chang et al., 2017). Double-stranded DNA damage activates ATM/ATR protein kinase, which activates P53 protein and DNA-dependent kinase (DNA-PKcs) and participates in the NHEJ pathway (Finzel et al., 2016; Menolfi and Zha, 2020). Ku70/80 recognizes and binds the double-stranded DNA ends, which attract DNA-PKcs to form new DNA-PK complexes. DNA-PK complexes continue to recruit NHEJ repair factors XRCC4, XLF, and DNA ligase IV to complete the repair of damaged DNA strands (Reynolds et al., 2012; Yue et al., 2020). The P53 protein enhances the catalytic activity of DNA ligase IV and promotes the NHEJ pathway (Huang and Zhou, 2021). Inhibition of core factors (Ku70/80 and DNA-PK) may lead to defects in the NHEJ pathway, severely affecting the repair of double-stranded DNA damage and possibly sensitizing tumor cells to various therapeutic approaches (Weterings et al., 2016; Chang et al., 2017).

## 3 Preclinical and clinical trials of DDR inhibitors in combination with ICIs in UC

### 3.1 DDR inhibitors

DDR inhibitors are a class of drugs that can interfere with or block the DDR process and have recently gained attention in cancer therapy research. The known DDR inhibitors can be classified into PARP inhibitors, ATM inhibitors, ATR inhibitors, CHK1/2 inhibitors, CDK4/6 inhibitors, WEE1 inhibitors, and DNA-PK inhibitors based on their targeting of different DNA repair pathways or factors (Cheng et al., 2022). The FDA has approved the PARP inhibitors olaparib, rucaparib, and niraparib for treating ovarian and breast cancers with germline BRCA gene mutations (Rose et al., 2020; Smith and Pothuri, 2022).

### 3.2 Clinical trial analysis of DDR inhibitors in combination with ICIs in UC

**FIGURE 2:**
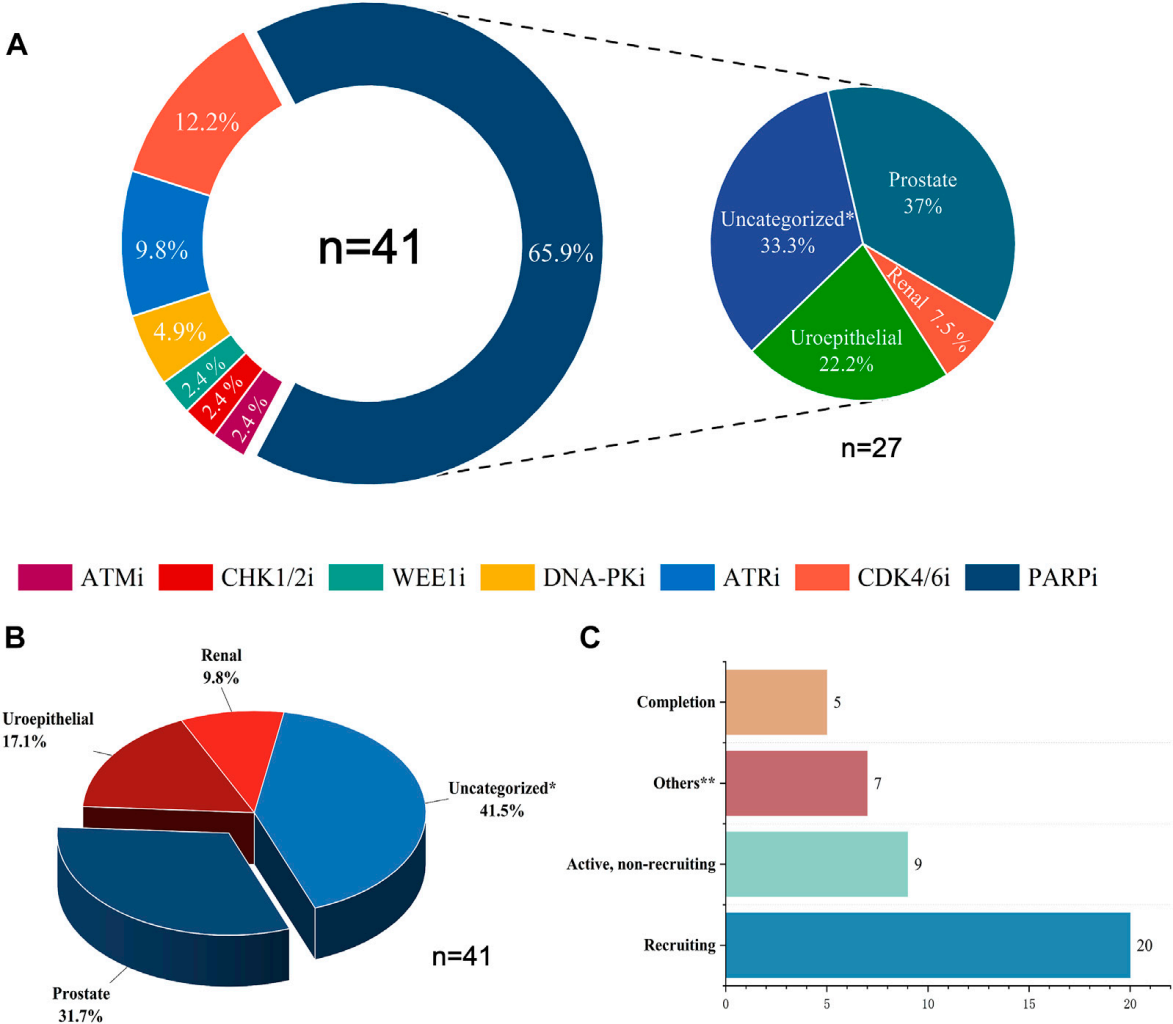
Statistical analysis of clinical trials of DDR inhibitors combined with ICIs for treating UC. **(A)** The distribution of inhibitors across 41 clinical trials and the distribution of UC types across 27 PARP inhibitor clinical trials. **(B)** Proportions of various UC types in 41 clinical trials. **(C)** Current status statistics for 41 clinical trials. Tips: Uncategorized* represents solid tumors not clearly classified, including urological malignancies; Uroepithelial includes solid tumors occurring in the uroepithelium (bladder, ureter, and renal pelvis); Others** include clinical trials that are currently terminated, not yet recruited, or have unknown status.

Combining DDR inhibitors with ICIs is effective for treating solid tumors such as ovarian and breast cancers, enhancing the effect of immunotherapy (Pilger et al., 2021). However, in UC treatment, although some preliminary findings have shown the potential therapeutic effect and clinical application of DDR inhibitors, studies on the combination of DDR inhibitors with ICIs are still in the initial stages. The mechanisms, efficacy, and safety of different combination therapies of DDR inhibitors and ICIs are still unclear (Cheng et al., 2022). We searched the CTG database (https://clinicaltrials.gov) and identified 41 clinical trials on the combination of DDR inhibitors and ICIs in UC and analyzed the current status of these trials (Figure 2). The analysis showed that 31.7% of the current clinical trials were in patients with prostate tumors, with a relatively low proportion of patients with other types of UC. In addition, 41.5% of the clinical trials were in patients with unspecified solid tumors, most were in the recruitment phase, and their recruitment criteria included patients with UC. Among the seven types of DDR inhibitors, clinical trials related to PARP inhibitors were the most popular, accounting for 65.9%, followed by CDK4/6 inhibitors, accounting for 12.2%, and other DDR inhibitors with ICIs accounted for a lower percentage of clinical trials. Of the 41 clinical trials, 20 recruited relevant patients, and only five have been completed and published. Research on the combination of DDR inhibitors and ICIs in UC has a long way to go, and the publication of the results of these trials may offer new options for immunotherapy in patients with UC.

### 3.3 Poly ADP-ribose polymerase (PARP) inhibitors

#### 3.3.1 PARP

PARP is a protein family consisting of 18 homologous domains. PARP-1 is the most typical representative of the PARP family and has a diverse function. Its structure includes DNA-binding, auto-polymerase, and regulatory domains (Curtin and Szabo, 2020). The DNA-binding domain recognizes and binds SSB sites, catalyzing the breakdown of nicotinamide adenine dinucleotide (NAD+) into nicotinamide and ADP-ribose. PARP catalyzes the polymerization of ADP-ribose units through its catalytic domain to form poly ADP-ribose (PAR) (Thomas et al., 2019). During DDR, PARP-1 is involved in the BER pathway. PARP-1 recognizes and binds to SSB damage during BER and enhances the efficiency of DNA repair by recruiting the XRCC1 DNA repair protein, which cooperates with DNA ligase and DNA polymerase β to repair SSBs (Wei and Yu, 2016; Ray Chaudhuri and Nussenzweig, 2017). PARP inhibitors can impede SSB repair, contribute to DSB formation, and increase tumor cell damage. Simultaneously, the activity of the DDR pathway is inhibited, resulting in DNA double-strand breaks that cannot be repaired (Rose et al., 2020). The increased DNA damage and impaired repair induce apoptosis in tumor cells (Hopkins et al., 2022).

#### 3.3.2 Mechanism of PARP inhibitors in combination therapy with ICIs

PARP inhibitors are currently in great demand and being investigated worldwide, and several pharmaceutical companies are actively developing PARP inhibitors and conducting extensive preclinical and clinical trials related to UC (Table 1). These include olaparib, developed by AstraZeneca; talazoparib, developed by Medivation and BioMarin; and pamiparib, developed by BGNE (Ashworth and Lord, 2018; Murthy and Muggia, 2019). Preclinical studies have shown that olaparib in tumor cells can increase DSB and promote apoptosis of tumor cells (Chen et al., 2021; Seo et al., 2022). PARP inhibitors induce cytoplasmic chromatin fragmentation with micronuclei formation, which can activate the cGAS-STING signaling pathway. The phosphorylation levels of the downstream effectors TBK1 and IRF3 were increased simultaneously (Chabanon et al., 2019; Zhang et al., 2022a). Activation of the cGAS-STING-TBK1-IRF3 signaling pathway promotes the expression of the downstream cytokine type I interferon (IFN) and chemokines such as CCL5 and CXCL10, which leads to T-cell recruitment while enhancing the infiltration and activation of cytotoxic T lymphocytes (Meng et al., 2021). In addition, increased IFN-β expression is also a direct mechanism of PD-L1 elevation by the ability of type I IFN to bind to IFN receptors on macrophages, activating the downstream JAK1/STAT signaling pathway and increasing PD-L1 expression (Meng et al., 2021; Wang et al., 2021). Another preclinical trial showed that PARP inhibitors inactivated GSK3β and prevented ubiquitination and degradation of PD-L1, further increasing PD-L1 expression (Jiao et al., 2017; Zhang et al., 2022a). In addition to these two mechanisms, another preclinical trial suggested that the application of PARP inhibitors increases PD-L1 expression by inhibiting the binding of miR-513 to PD-L1 (Sun et al., 2022). PARP inhibitors have also been shown to upregulate PD-L1 stability and expression by promoting the phosphorylation of CHK1 (Xue et al., 2020). Regardless of the specific mechanism mediating the upregulation of PD-L1 expression in tumors, the combination with ICIs further enhances the immune response and improves the efficacy of immunotherapy. The application of PARP inhibitors promotes immunogenic cell death, activates a robust immune response, increases the number and function of cytotoxic T lymphocytes, and causes more tumor cell death (Zhang et al., 2022a). In addition, PARP inhibitors reduced MDSC and immunosuppression-related Tregs in tumor tissue, spleen, and blood, which reversed the immunosuppressive TME and contributed to resensitizing tumor cells to immunotherapy (Ding et al., 2018). However, PARP inhibitors induce drug resistance as they activate the STAT3 signaling pathway and promote the shift of macrophages to the M2 type, which enhances immune escape and drug resistance in tumors. Therefore, combining PARP inhibitors with other drugs that inhibit the polarization of M2-type macrophages may enhance tumor cells’ sensitivity and immune response to PARP inhibitors (Ding et al., 2023). PARP inhibitor application increased the expression of ATR protein and contributed to the restoration of DDR function in tumor cells. The combination of ATR inhibitors may show a synergistic anti-proliferative effect (Nam et al., 2021). In addition, insufficient DNA damage caused by olaparib may activate the Wnt signaling pathway and mediate the development of drug resistance. In contrast, combining CDK4/6 inhibitors can cause cell arrest in the G1 phase to increase olaparib-induced DNA damage in the G2 phase, strongly inhibit olaparib resistance, and improve anti-tumor efficacy (Zhu et al., 2021a).

**TABLE 1:** Clinical trials of combination of PARP inhibitors and ICIs for UC.

DDR inhibitors	ICIs	Type of cancer	Stage	Clinical endpoints	Status	Clinical trial number
Niraparib	Atezolizumab	Uroepithelial carcinoma**	Ⅰ/Ⅱ	ORR, pCR, PFS, OS, DOR, DCR	Recruiting	NCT03869190
	Cetrelimab	Prostate Cancer	Ⅰ/Ⅱ	ORR, AEs, OS	Active, non-recruiting	NCT03431350
	Cetrelimab	Prostate Cancer	II	PFS, OS, AEs	Recruiting	NCT04592237
	Dostarlimab	Uncategorized*	II	ORR	Recruiting	NCT04779151
	Dostarlimab	Uncategorized*	II	ORR, PFS, OS, DOR, DCR	Recruiting	NCT05526989
Olaparib	Durvalumab	Prostate Cancer	II	Number of participants with undetectable PSA	Active, non-recruiting	NCT03810105
	Durvalumab	Uroepithelial carcinoma**	II	pCRR, AEs	Completion	NCT03534492
	Durvalumab	Prostate Cancer	II	Number of participants with undetectable PSA, AEs	Recruiting	NCT04336943
	Durvalumab	Uroepithelial carcinoma**	II	PFS, OS, ORR, DOR	Active, non-recruiting	NCT03459846
	Durvalumab	Uroepithelial carcinoma**	II	pCR, PFS, OS, AEs, ORR	Termination	NCT04579133
	Durvalumab	Renal cell carcinoma	II	AEs, tumor response	Recruiting	NCT03741426
	Durvalumab	Prostate Cancer	Ⅰ/Ⅱ	ORR, AEs	Recruiting	NCT02484404
	Durvalumab	Uroepithelial carcinoma**	I	ORR, DOR, PFS, OS, AEs, DCR	Active, non-recruiting	NCT02546661
	Durvalumab/Tremelimumab	Uncategorized*	II	PFS	Recruiting	NCT04169841
	Pembrolizumab	Prostate Cancer	II	ORR, PFS	Not yet recruited	NCT05568550
	Pembrolizumab	Prostate Cancer	III	OS, rPFS, ORR, DOR, TTP, AEs, TFST	Active, non-recruiting	NCT03834519
	Pembrolizumab	Uncategorized*	II	ORR, PFS, OS, AEs	Recruiting	NCT04123366
	Pembrolizumab	Prostate Cancer	Ⅰ/Ⅱ	Percentage of subjects with ≥50% decrease in PSA, AEs, ORR, OS, rPFS	Recruiting	NCT02861573
Pamiparib	Tislelizumab	Uncategorized*	I	AEs, DLT, ORR, PFS, DOR, DCR, CBR, OS	Completion	NCT02660034
	Tislelizumab	Uncategorized*	III	OS, AEs	Recruiting	NCT04164199
	Tislelizumab	Uncategorized*	II	CBR, ORR, PFS, OS, AEs	Recruiting	NCT04985721
Rucaparib	Atezolizumab	Uncategorized*	II	ORR	Termination	NCT04276376
	Nivolumab	Prostate Cancer	Ⅰ/Ⅱ	DLT, TTP, ORR	Termination	NCT03572478
	Nivolumab	Prostate Cancer	II	ORR, RR-PSA, rPFS, TTR, DOR, TTP,OS, AEs	Active, non-recruiting	NCT03338790
Talazoparib	Avelumab	Uncategorized*	II	DLT, ORR, Cmax, TTR, DOR, PFS, OS	Completion	NCT03330405
	Avelumab	Renal cell carcinoma	II	ORR, PFS	Active, non-recruiting	NCT04068831
	Avelumab	Uroepithelial carcinoma**	II	PFS, OS, DOR	Recruiting	NCT04678362

Tips: Uncategorized* represents solid tumors not clearly classified, including urological malignancies; Uroepithelial carcinoma** includes solid tumors occurring in the uroepithelium (bladder, ureter, renal pelvis).

Abbreviations: ORR, objective remission rate; AEs, frequency of adverse events; OS, overall survival; PFS, progression-free survival; pCR/PCRR, pathologic complete remission rate; DOR, duration of remission; DCR, disease control rate; DLT, dose-limiting toxicity; TTP, time to disease progression; RR-PSA, prostate-specific antigen response rate; rPFS, radiologic progression-free survival; TTR, time to tumor recurrence; PSA, prostate-specific antigen; TFST, time to next treatment; CBR, clinical benefit rate; Cmax, maximum observed blood concentration.

#### 3.3.3 Clinical trials of PARP inhibitors in combination with ICIs in UC

The CTG database (https://clinicaltrials.gov) currently has 27 clinical trials of PARP inhibitors in combination with ICIs related to UC actively underway, 37% of which focus on prostate cancer, 33.3% on solid tumors of unspecified classification (including UC), 22.2% on uroepithelial cancer, and 7.5% on renal cancer (Figure 2). NCT02854436 is a phase II clinical trial of niraparib as a single agent with the clinical endpoint of patient overall response rate (ORR) to assess the efficacy and safety of niraparib in patients with prostate tumors. The results showed an ORR of 34.2%, with more than 50% of patients experiencing adverse effects such as nausea and anemia and a small number of patients experiencing hematologic events such as thrombocytopenia and neutropenia. However, it is encouraging to note that the adverse effects of niraparib were tolerated by patients and that niraparib demonstrated antitumor activity against prostate tumors, especially in patients with BRCA mutations (Smith et al., 2022). Based on the promising performance of niraparib in UC, several phase I/II clinical trials are underway or enrolling patients with UC. Among them is NCT03869190, enrolling patients with uroepithelial tumors and combining niraparib with the ICI atezolizumab with the clinical endpoint of patient ORR to assess the efficacy and safety of this combination therapy. NCT03431350 and NCT04592237 are two ongoing clinical trials of niraparib in combination with ICI cetrelimab in prostate tumors, with the clinical endpoints of patient ORR, adverse events (AEs), and progression-free survival (PFS). Two clinical trials of niraparib in combination with ICI dostarlimab in solid tumors, urothelial carcinoma, and renal tumors are ongoing. The results of these trials may provide a basis for further clinical use of niraparib.

NCT03534492, NCT03459846, and NCT02484404 are three phase II clinical trials combining the PARP inhibitor olaparib with the ICI durvalumab in the treatment of urothelial carcinoma and prostate cancer. The clinical trial results in urothelial carcinoma showed a 44.5% complete remission rate in patients who underwent resection for muscle-invasive bladder cancer (MIBC). Further investigation revealed that patients with urothelial carcinoma who received the combination therapy had a PFS of 4.2 months compared to those treated with a single immunosuppressive agent (3.5 months) with an ORR of 28.2% and 18.4%, respectively. Nearly 50% of patients with uroepithelial cancer experienced adverse effects, including anemia and neutropenia. The trial data showed that the combination therapy demonstrated potent antitumor activity and improved patient survival. However, it caused some adverse effects that patients tolerated. This combination therapy has also shown promising results in prostate tumors. Clinical trial data indicate that the combination therapy is well tolerated by patients with prostate cancer, with only a small number of patients experiencing nausea. The combination therapy was efficacious in prostate tumors, with a median PFS of 16.1 months (95% CI: 4.5–16.1 months) for all patients, and surprisingly, it was more efficacious in men with DDR gene abnormalities, with a median PFS of 16.1 months (95% CI: 7.8–18.1 months) (Karzai et al., 2018). Six phase I/II clinical trials combining olaparib with durvalumab in prostate, urothelial, renal cell, and solid tumors are still ongoing or under recruitment (NCT03810105, NCT04336943, NCT04579133, NCT03741426, NCT02546661, and NCT04169841); these are designed to explore the efficacy and safety of this combination therapy in the treatment of different types of UC. In addition, four phase I/II/III clinical trials (NCT05568550, NCT03834519, NCT04123366, and NCT02861573) of olaparib in combination with pembrolizumab are actively underway to further explore the clinical results of this combination in the treatment of prostate tumors, and may provide a new option for patients with prostate tumors.

Several ongoing clinical trials of pamiparib in combination with ICI tislelizumab in UC. NCT02660034 is a phase I clinical trial of this combination therapy in prostate, uroepithelial, and other solid tumors. Nausea, fatigue, diarrhea, and vomiting were the most common adverse effects, with anemia being the most serious adverse effect with an ORR of 20%. The adverse drug reactions of this combination therapy were tolerated by patients with prostate, uroepithelial, and other solid tumors. It has some anti-tumor activity, and its clinical application in UC and other solid tumors deserves more in-depth exploration (Friedlander et al., 2019). NCT04164199 and NCT04985721 are two phase II/III clinical trials combining pamiparib with tislelizumab in solid tumors. These trials are currently in the patient recruitment phase, aiming to evaluate the efficacy and safety of this combination therapy in the treatment of advanced malignant solid tumors and provide a scientific basis for its clinical application.

NCT03330405 is a phase II clinical trial combining the PARP inhibitor talazoparib and ICI avelumab in advanced solid tumors. The trial results showed that 11.1% of patients with DDR-deficient prostate tumors had partial remission, and no patients had complete remission after the combination therapy. In contrast, none of the DDR-perfect patients had remissions of symptoms. For patients with DDR-deficient prostate cancer, the PFS was 4.6 months longer than for patients with DDR-perfect prostate cancer. In contrast, for patients with uroepithelial tumors, the combination therapy led to complete remission in 2.5% and partial remission in 12.5%, with a PFS of 3.6 months. The most common adverse effects were anemia, thrombocytopenia, and neutropenia. In this clinical trial, the combination therapy was effective in patients with DDR-deficient prostate and uroepithelial tumors, and the patients tolerated the toxic effects. It also suggests that it is crucial to select the correct type of patients for combination therapy with ICI and PARP inhibitors (Yap et al., 2023). Two phase II trials combining talazoparib and avelumab in renal and uroepithelial tumors are ongoing or enrolling patients (NCT04068831, NCT04678362), and the results of these trials may guide the dosage and considerations of this combination therapy in UC treatment in the future.

NCT03397394 was a phase II clinical trial of the PARP inhibitor rucaparib as a single agent in uroepithelial tumors. However, the trial results were discouraging. Rucaparib did not show significant activity in treating patients with advanced uroepithelial carcinoma, regardless of whether these patients had mutations in homologous recombination repair (HRR) (Grivas et al., 2021). However, promising results were achieved in a phase II clinical trial of rucaparib combined with nivolumab for treating prostate tumors (NCT03338790). The trial results showed that the combination was highly effective in patients with HRRd-positive prostate tumors, especially those carrying BRCA1/2 mutations, and that the adverse effects of the therapy, which included mainly nausea and anemia, were well tolerated by patients (Fizazi et al., 2022). In addition, two phase I/II clinical trials combining rucaparib with atezolizumab and nivolumab in solid and prostate tumors, respectively, have been discontinued due to lack of funding or lack of efficacy (NCT04276376, NCT03572478). Therefore, the clinical efficacy and safety of the combination of rucaparib and ICI in treating UC are unclear. More clinical trials are needed to further explore this potential combination therapy in the clinical application of UC.

### 3.4 Ataxia-Telangiectasia mutated (ATM) kinase inhibitors

#### 3.4.1 ATM

ATM is a member of the phosphatidylinositol 3-kinase (PI3K)-related protein kinase (PIKK) family, located at 11q22.3. ATM can regulate cell cycle checkpoints, including G1/S and G2/M arrest and induction of apoptosis. Moreover, ATM plays a vital role in detection and signal transduction in the DDR process (Jin and Oh, 2019). In response to DNA damage such as DSBs, ATM is activated to form macromolecular aggregates with the MRN complex consisting of MRE11, RAD50, and NBS1 at DSBs and phosphorylates a series of downstream signal transduction proteins (Lee and Paull, 2005). ATM phosphorylates the downstream kinase CHK2 and activates proteins such as P53, BRCA1, and RAD51/52 to facilitate HRR repair. ATM also inhibits the NHEJ repair pathway to avoid misrepair (Zhang et al., 2022b; Qi et al., 2022). Furthermore, ATM phosphorylates histone H2AX molecules at serine 139, transforming them into γ-H2AX, which forms foci at DNA damage sites during DDR, recruiting more DDR proteins to the damage site and facilitating efficient DDR (Pan et al., 2011).

#### 3.4.2 Mechanism of combination therapy of ATM inhibitors and ICIs

Many preclinical trials have been conducted to explore the mechanism of the combined application of ATM inhibitors and ICIs to enhance the efficacy of immunotherapy. The application of ATM inhibitors to tumor cells blocks ATM signaling in tumor cells, leading to the inability to activate the G1/S cell cycle checkpoint and massive tumor cell death (Chiu et al., 2023). ATM inhibitors lead to defective DNA damage repair, which in turn induces chromosomal instability and abnormal division, forming micronuclei. Micronuclei contain unrepaired DNA breaks or circular DNA that can be recognized by cGAS in the cytoplasm and activate the STING/TBK1 signaling pathway, thereby upregulating type I IFN expression and enhancing inflammatory signaling (Chiu et al., 2023). In addition, ATM inhibitors affect mitochondrial function and number and downregulate transcription factors such as TFAM. They also lead to the release of mitochondrial DNA from the mitochondria into the cytoplasm, enhancing lymphocyte infiltration in TME through the cGAS/STING signaling pathway (Hu et al., 2021). A preclinical trial have confirmed the negative correlation of ATM expression with PD-L1 expression and that type I IFN binds to IFN receptors on macrophages, activating downstream JAK1 as well as STAT1/3 signaling. This leads to increased PD-L1 expression and enhanced sensitivity of tumor cells to anti-PD-L1/PD-1 antibodies, which may be associated with increased infiltration of CD8^+^ T-cells in TME; the construction of immune memory is closely related (Zhang et al., 2019; Gao et al., 2023). Further exploration of ATM inhibitors confirmed that ATM inhibition may upregulate Gal-9 expression through the cGAS-STING-IFN-β signaling pathway, an important mechanism mediating tumor immune escape. Combination with Gal-9 inhibitors could further enhance the efficacy of ATM inhibitors and alter the efficacy of those anti-PD-1/PD-L1 malignancies that are tolerant to immunotherapy, providing a new way to improve immunotherapy in UC and solid tumors (Zheng et al., 2023).

#### 3.4.3 Clinical trials of combination therapy with ATM inhibitors and ICIs in UC

A multicenter clinical trial explored the impact of DDR gene alterations on OS in patients with advanced uroepithelial carcinoma treated with anti-PD-1/PD-L1 immunotherapy, with the clinical endpoints of ORR and overall survival (OS). The effect may be attributed to the deletion of ATM protein, leading to higher TMB, and tumor cells with high TMB expression may have more neoantigens and therefore be more sensitive to immunotherapy. However, this experiment also found that although altered ATM improved patients’ sensitivity to immunotherapy, OS was not promising, probably because ATM plays multiple roles in cancer development and changes in its protein expression could accelerate the process of epithelial-mesenchymal transition (EMT) in some tumor cells, leading to a poor prognosis (Joshi et al., 2020). The exact mechanism by which ATM inhibition leads to reduced OS is not yet understood. Many clinical trials are exploring the efficacy and safety of ATM deficiency in treating solid tumors with ATM inhibitors. In addition, a Phase I clinical trial (NCT05396833) of the ATM inhibitor M4076 combined with avelumab for treating advanced solid tumors is in the recruitment stage, and the clinical endpoints are dose-limiting toxicity (DLT) and AEs. The results of this clinical trial may provide additional clinical data to support the widespread use of ATM inhibitors (Table 2).

**TABLE 2:** Clinical trials of other DDR inhibitors and ICIs in combination for UC.

DDR inhibitor type	DDR inhibitors	ICIs	Type of cancer	Stage	Clinical endpoints	Status	Clinical trial number
ATM inhibitors	M4076	Avelumab	Uncategorized*	I	DLT, AEs	Recruiting	NCT05396833
ATR inhibitor	Berzosertib	Avelumab	Uncategorized*	Ⅰ/Ⅱ	AEs, DLT, PFS, OS, CBR, MTD	Recruiting	NCT04266912
	Ceralasertib	Durvalumab	Uncategorized*	II	ORR, DOR, PFS, AEs	Recruiting	NCT03682289
		Durvalumab	Uncategorized*	I	DLT, ORR, PFS, DOR	Recruiting	NCT05514132
	M1774	Avelumab	Uncategorized*	I	DLT, AEs, ORR	Recruiting	NCT05396833
CHK1/2 inhibitor	prexasertib	LY3300054	Uncategorized*	I	DLT, Cmax	Completion	NCT03495323
CDK4/6 inhibitors	Abemaciclib	Atezolizumab	Prostate Cancer	II	PFS, ORR, DLT, AEs, CBR, DOR, DOT, TTP, OS	Recruiting	NCT04751929
		Atezolizumab	Prostate Cancer	II	PFS, DLT, ORR, CBR, DOR, DOT, TTP, OS, AEs	Unknown	NCT04272645
	Palbocicilib	Sasanlimab	Renal clear cell carcinoma of the kidney	Ⅰ/Ⅱ	DLT, ORR, DCR, PFS, OS	Not yet recruited	NCT05665361
		Avelumab	Renal clear cell carcinoma of the kidney	II	ORR, PFS, OS	Not yet recruited	NCT05176288
	Trilaciclib	Avelumab	Uroepithelial carcinoma**	II	PFS, ORR	Active, non-recruiting	NCT04887831
WEE1 inhibitor	AZD1775	Durvalumab	Uncategorized*	I	DLT, AEs, ORR, PFS, DCR, OS	Active, non-recruiting	NCT02617277
DNA-Pk inhibitor	M3814	Avelumab	Uncategorized*	I	DLT, Cmax, AEs, DOR, PFS, OS	Completion	NCT03724890
		Avelumab	Prostate Cancer	Ⅰ/Ⅱ	DLT, rPFS, PFS, OS, AEs	Recruiting	NCT04071236

Tips: Uncategorized* represents solid tumors not clearly classified, including urological malignancies; Uroepithelial carcinoma** includes solid tumors occurring in the uroepithelium (bladder, ureter, renal pelvis).

Abbreviations: DLT, dose limiting toxicity; AEs, frequency of adverse events; ORR, objective remission rate; PFS, progression-free survival; DCR, disease control rate; OS, overall survival; DOR, duration of remission; CBR, clinical benefit rate; MTD, maximum tolerated dose; DOT, duration of treatment; TTP, time to disease progression Cmax, maximum observed blood concentration; rPFS, radiological progression-free survival.

### 3.5 Ataxia-Telangiectasia and Rad3-Related protein (ATR) inhibitors

#### 3.5.1 ATR

ATR is another member of the PIKK family that prevents cells from entering the cell cycle when subjected to DNA damage and ensures the integrity and stability of DNA (Blackford and Jackson, 2017). ATR is important in the DDR process, interacting with ATRIP to sense ssDNA damage while phosphorylating and activating downstream effectors, interacting with TOPBP1 to activate CHK1, and promoting DNA damage repair together with BRCA1 (Gralewska et al., 2020). Inhibition of ATR expression interferes with the response of tumor cells to DNA damage and makes them more susceptible to therapies such as radiotherapy, chemotherapy, and immunotherapy (Fokas et al., 2014).

#### 3.5.2 Mechanism of ATR inhibitors in combination with ICIs

Several pharmaceutical companies have developed specific ATR small molecule inhibitors and are conducting preclinical and clinical trials related to urologic malignancies (Table 2), including AstraZeneca’s berzosertib (M6620), Selleck’s AZD6738, and Bayer’s BAY1895344 (Barnieh et al., 2021). Preclinical trials with the combination of BAY1895344 and anti-PD-L1 antibodies in prostate tumors confirmed that the combination of the two drugs produced synergistic antitumor activity and further improved the efficacy of immunotherapy. ATR inhibitors inhibited the ATR-CHK1-CDK1-regulated G2/M cell cycle checkpoint and promoted prostate tumor cell death and activation of the cGAS-STING signaling pathway. The activated cGAS-STING signaling pathway promoted IFN-β signaling in TME, along with increased expression of IFN-inducible genes (ISG), including CCL5 and CXCL10(97). Increased expression of IFN-β and ISG enhances the function of natural and adaptive immunity, improves the body’s immune surveillance ability against prostate tumors, and increases the effectiveness of immunotherapy (Yu et al., 2022). In addition, ATR inhibitors induced an unstable state of PD-L1 in prostate tumor cells. This may be attributed to the inhibition of the ATR-CHK1 signaling pathway, leading to the activation of the CDK1-SPOP axis, affecting the function of ubiquitin ligases, promoting the ubiquitination and degradation of PD-L1, and thus reducing its expression level (Tang et al., 2021). PD-L1/PD-1 interaction can suppress T-cell activation and function by inhibiting the PI3K-AKT-mTOR signaling pathway downstream of the T-cell receptor and reducing T-cell proliferation, differentiation, and effector molecule production. In contrast, downregulation of PD-L1 expression attenuates PD-L1/PD-1 interaction and further enhances T-cell immune responses (Sun et al., 2018a). In addition, downregulation of PD-L1 expression also activated the IFNAR1-JAK1 signaling pathway, promoted phosphorylation of STAT3 at Y705 and cleavage of caspases 3/7, induced the IFN-β-mediated cytotoxic pathway, and promoted apoptosis in prostate tumor cells (Tang et al., 2021). Another preclinical trial investigated the efficacy of berzosertib in combination with anti-PD-L1 antibodies in malignant tumors. The results suggested that combination therapy increased the infiltration of CD8^+^ T-cells and improved anti-tumor efficacy. In addition to the conventional modulation of cell cycle checkpoint G2/M to promote apoptosis, ATR inhibitors activate the cGAS-STING-TBK1/IRF3 axis, a classical STING signaling pathway, by increasing the level of cytoplasmic double-stranded DNA (dsDNA). At the same time, ATR inhibitors also activate non-classical STING signaling by promoting SUMO-ization of SHP1 at lysine 127 and attenuating SHP1-mediated inhibition of the TRAF6-STING-p65 signaling pathway. Both classical and non-classical STING signaling pathways promoted type I IFN expression, induced activation of innate immunity, reversed immunosuppressive TME, and further improved the efficacy of immunotherapy (Liu et al., 2023).

#### 3.5.3 Clinical trials of ATR inhibitors in combination with ICIs in UC

NCT02567409 is an ongoing phase II clinical trial of the ATR inhibitor berzosertib combined with chemotherapeutic agents in patients with uroepithelial bladder cancer, with the clinical endpoints of PFS and OS. Berzosertib was associated with a shorter median PFS and OS of 19.8 months and 14.4 months, respectively, in patients with metastatic bladder cancer compared to those treated with chemotherapy alone. In addition, patients with the combination of berzosertib had a higher rate of serious adverse reactions than those with the single chemotherapy regimen, mainly thrombocytopenia and neutropenia. This suggests that berzosertib may have high hematologic toxicity and that more clinical trials are needed to explore the safety and dosing regimen of berzosertib in combination with UC (Pal et al., 2021). NCT03517969 is a phase II clinical trial of berzosertib in combination with chemotherapeutic agents in prostate cancer patients, with ORR and PFS as clinical endpoints. The results may provide a new basis for the safety and efficacy of berzosertib in the combination treatment of urologic malignancies. In addition, clinical trials are actively underway combining ATR inhibitors with ICIs in UC. NCT03682289 and NCT05514132 are phase I/II clinical trials combining the ATR inhibitor ceralasertib with the ICI durvalumab in solid tumors, whereas NCT04266912 and NCT05396833 are phase I/II clinical trials combining berzosertib with M1774 and avelumab in solid tumors. The above four clinical trials are in the recruitment phase. Their clinical endpoints are mainly DLT, ORR, and PFS, aiming to evaluate the efficacy and safety of the combination therapies. The publication of future results may bring new hope to patients with UC who are tolerant to immunotherapy.

### 3.6 Checkpoint kinase 1/2 (CHK1/2) inhibitors

#### 3.6.1 CHK1/2

CHK1/2 is an important serine/threonine protein kinase in cellular DNA damage response and cell cycle regulation and is a downstream target of ATR/ATM. In response to DNA damage, ATR/ATM kinase is activated and phosphorylates CHK1/2, thereby stimulating its activity. CHK1 directly phosphorylates and inhibits the activity of CDC25A/C, thereby preventing the activation of the CDK1/Cyclin B complex and leading to G2/M phase block. CHK2 directly phosphorylates and stabilizes P53 protein, thereby promoting P21 expression and inhibiting the activation of the CDK2/Cyclin E complex, leading to G1/S phase block. These behaviors ensure that DNA has sufficient time to undergo repair (Vincelette et al., 2019). CHK1/2 also promotes the activity of the HRR pathway, inhibits the activity of DNA-PK, reduces the frequency of the NHEJ pathway, and avoids the occurrence of DNA repair errors (Carlsen and El-Deiry, 2022).

#### 3.6.2 Mechanism of CHK1/2 inhibitor combination therapy with ICIs

High CHK1 expression may be associated with poor tumor prognosis, and inhibition of CHK1/2 expression by CHK1 inhibitors or other related factors (e.g., IRF-1, oxidative stress) can lead to the accumulation of DNA damage by blocking the DDR process while increasing the number of infiltrating NK cells, which promotes apoptosis of tumor cells. In addition, inhibition of CHK1 increased STAT3 phosphorylation and further upregulated PD-L1 expression (Ding et al., 2016; Yan et al., 2021). This suggests that inhibition of CHK1/2 may decreases the resistance of tumor cells to ICB immunotherapy. Therefore, combining CHK1/2 inhibitors with ICIs is being explored as a promising antitumor therapy, and relevant preclinical studies and clinical trials are actively underway (Table 2).

Currently, there are no preclinical studies related to urological malignancies; however, preclinical studies on small cell lung cancer (SCLC) may provide some theoretical basis. Because both SCLC and urologic malignancies (such as prostate cancer and bladder cancer are highly proliferative, prone to DNA damage, and highly express CHK1, they may respond similarly to CHK1/2 inhibitors in combination with ICIs (Doerr et al., 2017; Zheng et al., 2018; Twomey and Zhang, 2021).

*In vitro* application of the CHK1 inhibitor prexasertib to SCLC cells revealed an increase in cytosolic DNA and activation of the cGAS-STING-TBK1 signaling pathway, a significant increase in PD-L1 expression, and suppression of tumor immune escape. The application of CHK1 inhibitors also activated the STING-TBK1-IRF3 signaling pathway and increased the expression of IFN-β, while the expression levels of immune chemokines CXCL10 and CCL5 were also significantly increased. The immunogenicity of tumor cells was enhanced, cytotoxic T lymphocytes were activated and participated in the biological process of killing tumor cells, the efficacy of ICIs in some tumor cells was greatly enhanced, and the drug resistance response was significantly reversed (Sen et al., 2019a). In another preclinical study of SCLC, the combination of the CHK1 inhibitor SRA737 with anti-PD-L1 increased the M1 subtype macrophage population, decreased the expression of the immunosuppressive myeloid suppressor cell (MDSC) population, and greatly improved the immune microenvironment of tumor cells, further enhancing the therapeutic efficacy of ICB therapy (Sen et al., 2019b).

#### 3.6.3 Clinical trials of CHK1/2 inhibitors in combination with ICIs in UC

In clinical trials, various CHK1/2 inhibitors have been used as single agents or combined with chemotherapeutic agents and ICIs in urologic malignancies to explore their efficacy and safety in treating tumors. NCT02203513 was a phase II clinical trial using the CHK1 inhibitor LY2606368 as a single agent in prostate cancer. The clinical endpoints were ORR and AEs; however, the sponsor, Eli Lilly and Company, terminated the trial after failing to meet the clinical endpoints. Patients with prostate cancer experienced anemia, bloating, abdominal pain, diarrhea, nausea, and vomiting following the administration of LY2606368. Further investigations are needed to ascertain whether these reactions were due to drug therapy or cachexia related to the tumor. NCT03495323 is a phase I clinical trial of the CHK1 inhibitor prexasertib combined with the anti-PD-L1 antibody LY3300054 in solid tumors, with the clinical endpoints of DLT and AEs. The results showed that the combination of prexasertib and LY3300054 was tolerable during the human clinical trial, with fewer patients possibly experiencing fever and neutropenia as AEs. However, blood investigation results showed activation of cytotoxic T lymphocytes, suggesting that the combination of CHK1/2 inhibitors with ICIs could enhance the anti-tumor activity and reverse the tumor cells’ resistance to ICB therapy (Do et al., 2021). In addition, phase I/II clinical trials of the combination of CHK1/2 inhibitors SRA737 and AZD7762 with chemotherapeutic agents in solid tumors are actively underway (NCT02797977, NCT00413686), aiming to investigate the safety and efficacy of the combination of CHK1 inhibitors and chemotherapeutic agents in the treatment of solid tumors. The results could provide further evidence for the use of CHK1/2 inhibitors in urologic malignancies and solid tumors.

### 3.7 Cell cycle-dependent kinase 4/6 (CDK4/6) inhibitors

#### 3.7.1 CDK4/6

CDK4/6 is a class of serine/threonine kinases that binds to D-cyclins to form a complex that phosphorylates retinoblastoma protein (RB) and induces the release of transcription factor E2F from the transcriptional repressor complex Rb-E2F, promoting the entry of cells from G1 phase to S phase (Topacio et al., 2019). During DDR, CDK4/6 activity is inhibited, causing the cells to stagnate in the G1 phase. The mechanism is the regulation of CDK4/6 by P53 and p16INK4a proteins, with P53 promoting the expression of the CDK inhibitor P21, while p16INK4a directly binds CDK4/6 and recruits MDM2. This series of events inhibits the activity of CDK4/6, thus giving the cells enough time to complete the DNA damage repair process (Kulaberoglu et al., 2021). In addition, CDK4/6 can regulate ATM/ATR activity and initiate the DDR pathway (Hashizume et al., 2016). CDK4/6 inhibitors can block the cycle of tumor cells and inhibit their proliferation and growth. In contrast, CDK4/6 inhibitors can contribute to the death of many tumor cells by promoting the apoptotic pathway and modulating the immune system to halt the progression of tumor development (Goel et al., 2017; Goel et al., 2022).

#### 3.7.2 Mechanism of combination therapy with CDK4/6 inhibitors and ICIs

Several companies have developed CDK4/6 inhibitors, including Pfizer’s palbociclib, ribociclib, and Eli Lilly’s abemaciclib (Perez-Garcia et al., 2021). CDK4/6 inhibitors inhibit the CDK1-Cyclin D4 complex, which induces cell cycle arrest in the G1 phase and apoptosis in some tumor cells (Zhu et al., 2021b; Salewski et al., 2022). CDK4/6 inhibitors also increase PD-L1 expression. A preclinical study of prostate tumors showed that 10%–15% had SPOP mutations, and palbociclib promoted SPOP degradation by preventing phosphorylation of SPOP mediated by D-CDK4. Mutations in SPOP degradation inhibited ubiquitination-mediated PD-L1 degradation, PD-L1 expression was increased, and the effect of immunotherapy was further improved (Zhang et al., 2018; Zeng et al., 2021). Preclinical studies have shown that CDK4/6 inhibitors dephosphorylate RB, inhibit the activation of the transcriptional repressor complex RB-E2F, and downregulate the expression of the DNA methyltransferase DNMT1, enhancing the activation of cytotoxic cells and improving the antigen presentation of tumor cells (Goel et al., 2017; Schaer et al., 2018; Bai et al., 2023). CDK4/6 inhibitors activated endogenous retroviral elements, leading to increased dsDNA expression, promoting IFN expression, activating the body’s innate and adaptive anti-tumor immune response, and greatly improving the efficacy of immunotherapy (Tong et al., 2022).

The most important function of CDK4/6 inhibitors in treating malignancy is reprogramming TME (Jang et al., 2022). Immune infiltration in TME, including CD8^+^ T lymphocytes and B-cells, is further enhanced by applying CDK4/6 inhibitors to tumor cells (Zhang et al., 2020). The secretion of CXCL10 and CXCL13 was also promoted, and more lymphocytes were recruited. This may be due to enhanced antitumor cytotoxicity and increased expression of IFNγ along with the promotion of Th1 cytokine levels (Deng et al., 2018; Zhang et al., 2020). In addition, the findings suggest a high CDK6 expression in Tregs, which is strongly dependent on CDK6, and that the application of several CDK4/6 inhibitors reduces the expression of the immunosuppressive cell population in Tregs (Deng et al., 2018). Moreover, the expression of immunosuppressive cells (Tregs, MDSC) in TME was further reduced through the inhibition of NFAT family proteins and their target genes by CDK4/6 inhibitors (Yu et al., 2019; Zhang et al., 2020). Combining palbociclib with an anti-PD-1 antibody also promotes NK cell infiltration and CD107a expression in NK cells, increased infiltration of active cytotoxic cells, and a series of events that synergistically reverse the immunosuppressive TME, increasing the sensitivity of tumor cells to immunotherapy (Bai et al., 2023). CDK4/6 inhibitors also lower the apoptotic threshold of tumor cells by inhibiting the phosphorylation of P73, leading to nuclear translocation of P73, and inducing activation of DR5, allowing tumor cells to be readily affected by multiple therapeutic modalities (Tong et al., 2022). The activation of DR5 also promotes immunogenic cell death in some tumor cells, releasing many damage-associated molecules (DAMPs), including CRT, HMGB1, and ATP, further enhancing the immunogenicity of tumor cells (Teo et al., 2017; Tong et al., 2022).

#### 3.7.3 Clinical trials of CDK4/6 inhibitors in combination with ICIs in UC

NCT00003256 and NCT00016939 are two phase II clinical trials of alvocidib as a single agent in prostate cancer and renal cell carcinoma, respectively. The clinical trial results for prostate cancer showed that only 14% of patients on the CDK4 inhibitor alvocidib met the endpoint of 6-month PFS and the response to alvocidib single-agent activity in prostate tumors was disappointing; it should only be used in combination or as an alternative therapy (Liu et al., 2004). In contrast, the trial results in renal cell tumors were promising, with alvocidib having acceptable toxic adverse effects and efficacy in patients with advanced renal cell tumors: 12% of 34 patients experienced complete or partial remission, and 41% had stable disease with a median OS of 9 months. Adverse reactions to alvocidib were mainly diarrhea, vomiting, anemia, and dyspnea, nevertheless, the adverse effects were tolerable (Van Veldhuizen et al., 2005). Abemaciclib, developed by Eli Lilly and Company, is currently in two Phase I clinical trials (NCT03837821 and NCT04627064) in patients with uroepithelial bladder cancer and renal clear cell tumors, respectively. The clinical endpoints are ORR, PFS, and OS. NCT04887831 is an ongoing phase II trial of the CDK4/6 inhibitor trilaciclib in combination with the ICI avelumab in advanced uroepithelial bladder cancer, with the clinical endpoint of 7-month PFS in patients. In addition, NCT04751929 and NCT04272645 are two Phase II clinical trials of abemaciclib in combination with atezolizumab in prostate cancer, with clinical endpoints of 6-month PFS and DLT in patients. NCT04751929 is recruiting to evaluate the safety, efficacy, and adverse effects of the combination of abemaciclib and atezolizumab for prostate tumors. Two Phase I/II clinical trials (NCT05176288, NCT05665361) for renal clear cell tumors have not yet begun recruitment; these trials will investigate the CDK4/6 inhibitor palbociclib acting with the ICIs avelumab and sasanlimab, respectively. The clinical endpoint is patient ORR. The results of this series of clinical trials of CDK4/6 inhibitors in combination with ICIs may provide new ideas and options for immunotherapy in UC (Table 2).

### 3.8 WEE1 inhibitor

#### 3.8.1 WEE1

WEE1 is a tyrosine/threonine kinase that phosphorylates the threonine or tyrosine residues of CDK, inhibiting CDK activity and preventing cells from entering mitosis (Leijen et al., 2016). During DDR, WEE1 binds to the CDK1 complex. It inhibits CDK1 activity through phosphorylation, promoting cell cycle arrest in the G2/M phase, allowing sufficient time for complete DNA repair, and preventing incompletely repaired DNA from entering mitosis (Beck et al., 2012). Inhibition of WEE1 protein expression disrupts cell cycle regulation, increases DNA damage and genomic instability, and affects tumor cell development. At the same time, deleting the WEE1 protein increases the sensitivity of tumor cells to treatment and produces a synthetic lethal effect (Do et al., 2013).

#### 3.8.2 Mechanism of combination therapy with WEE1 inhibitors and ICIs

Several ongoing clinical trials of the WEE1 inhibitors, including ADZ5338, MK-1775, and PD0166285, reported limited progress. AZD1775, a WEE1 inhibitor developed by AstraZeneca, and used in preclinical studies and clinical trials for malignancies showed some progress (Do et al., 2015; Ghelli Luserna di Rorà et al., 2020). The application of WEE1 inhibitors to tumor cells inhibits the activation of the G2/M cell cycle checkpoint, forcing tumor cells with unrepaired DNA damage to enter prematurely into mitosis, and promotes granzyme B-induced CDK1 phosphorylation, a dual effect that enhances the killing effect of cytotoxic T lymphocytes on antigen-positive tumor cells, causing a large number of tumor cells to die (Sun et al., 2018b; Friedman et al., 2018; Patel et al., 2019). A preclinical study showed that AZD1775 inhibited the phosphorylation processes of CDC2 and CDC25C, significantly increasing apoptosis in sensitive tumor cells. However, increased expression of ATR and ATM proteins was observed in insensitive tumor cells. The increase in DDR-related proteins may promote tumor cell progression by other pathways in insensitive tumor cells. It is suggested that the combination of a WEE1 inhibitor and an ATR/ATM inhibitor can enhance the anti-tumor efficacy in insensitive tumor cells (Nam et al., 2020). Co-inhibition of WEE1 and ATM can reduce the expression of cytokines such as MMP-9 and IL-8, which are closely related to the migration and invasion abilities of tumor cells (Jin et al., 2020). In addition, the co-inhibition of WEE1 and ATM inhibited the activation of the FAK-Src-CREB signaling pathway, which is closely related to the migratory invasive ability of tumor cells. These multiple effects inhibited the migratory invasive ability of tumor cells, thereby enhancing their anti-tumor efficacy (Jin et al., 2020).

Reduced expression of PD-L1 was found after applying WEE1 and ATM inhibitors to tumor cells, which may result from reduced expression levels of CMTM6 and GSK-3β (Jin et al., 2020). CMTM6 binds PD-L1 and maintains its expression on the cell surface to avoid PD-L1 degradation by lysosomes. The regulation of CMTM6 involves genes such as BRCA1, CREB, and TEAD1, and WEE1 inhibitors decrease the expression of proteins such as CREB and possibly increase the expression of HIP1R to compete with CMTM6 for binding PD-L1. This further breaks the interaction between CMTM6 and PD-L1, contributing to the decrease in PD-L1 expression (Mezzadra et al., 2017; Jin et al., 2020; Jin et al., 2021). AZD1775 can also downregulate PD-L1 expression by inhibiting STAT3 phosphorylation and reducing IRF1 expression (Wang et al., 2020). Decreased PD-L1 expression attenuated the PD-L1/PD-1 interaction and further enhanced the immune response of T-cells (Sun et al., 2018a). In contrast, studies on ovarian tumors showed that WEE1 inhibitors promoted PD-L1 expression, recruited activated immune-related cells, and induced massive tumor cell death in a CD8^+^ T cell-dependent manner (Guo et al., 2022), which is attributed to the fact that WEE1 inhibitors further increase the expression of endogenous retrovirus (ERV) by downregulating FOXM1 to alleviate SETDB3/H9K3me1 inhibition. ERV activates dsDNA and IFN responses, promotes type I IFN expression even in STING-deficient tumor cells, enhances natural and adaptive immunity, and improves the effectiveness of immunotherapy (Guo et al., 2022). Applying WEE1 inhibitors to tumor cells revealed a significant reduction in the expression of M2 macrophages and Treg immunosuppressive cells in TME, reversing the suppressive TME and increasing the sensitivity of tumor cells to immunotherapy (Jin et al., 2021). This may be a result of the WEE1 inhibitor reducing the release of PAI-1 and blocking the recruitment and activation of M2 macrophages. WEE1 inhibitors also inhibited the release of the cytokine CCL6, which severely affected the recruitment, differentiation, and function of cell populations associated with Treg immunosuppression (Jin et al., 2021). In addition, WEE1 inhibitors significantly increased the expression of CRT, HMGB1, and other DAMPs in TME, which are closely associated with immunogenic cell death (ICD), suggesting that WEE1 inhibitors may further enhance the efficacy of immunotherapy and induce tumor cell death through ICD (Dinavahi et al., 2022). However, it is noteworthy that this effect requires the expression of P53, partly suggesting that P53 may be a predictive biomarker of the sensitivity of patients to WEE1 inhibitors (Dinavahi et al., 2022).

#### 3.8.3 Clinical trials of WEE1 inhibitors in combination with ICIs in UC

NCT01748825 was a phase I clinical trial of the WEE1 inhibitor AZD1775 for treating patients with advanced solid tumors. The clinical endpoint was DLT. The results showed that the most common toxicities were myelosuppression and diarrhea. The DLTs were supraventricular arrhythmias and myelosuppression. However, the hematologic and gastrointestinal toxicities were manageable and tolerable for treating solid tumors (Do et al., 2015). AstraZeneca is currently conducting a phase I clinical trial (NCT02617277) designed to evaluate the safety and tolerability of AZD1775 in combination with MEDI4736 (durvalumab) in advanced solid tumors with clinical endpoints of DLT, PFS, OS, and AEs. The trial results may further validate the use of this combination therapy in UC (Table 2).

### 3.9 DNA-dependent protein kinase (DNA-PK) inhibitors

#### 3.9.1 DNA-PK

DNA-dependent protein kinase is responsible for repairing DNA damage in cells. It consists of three subunits: DNA-PKcs, the DNA-PK catalytic subunit, and Ku70 and Ku80, the DNA-binding subunits that recognize DSBs during DNA repair. These form a complex with the broken DNA ends. This complex can recruit the DNA-PK catalytic subunit DNA-PKcs and activate its catalytic activity (Valikhani et al., 2021). Activated DNA-PKcs phosphorylates other repair proteins, such as ATM and BRCA1. DNA-PKcs participate in DDR processes with these repair proteins, including cell cycle regulation and activation of the NHEJ repair pathway (Bergstrand et al., 2022).

#### 3.9.2 Mechanism of combination therapy with DNA-PK inhibitors and ICIs

DNA-PK inhibitors can cause apoptosis of tumor cells by inhibiting the activity of DNA-PK kinase, which affects the process of NHEJ. The expression of PD-1 in TME was significantly reduced after applying DNA-PK inhibitors, and the combined application of anti-PD-1 antibodies further enhanced the immune system’s ability to attack tumor cells (Nakamura et al., 2021). In addition, the findings suggest that NK cell granzyme B expression is elevated in the TME and type I IFN signaling is activated. A series of changes in immune molecules prompts the body to generate tumor antigen-specific immune memory, which acts as a rapid immune response during tumor re-emergence (Nakamura et al., 2021). Another preclinical study on DNA-PK elucidated the mechanism of activation of type I IFN signaling in TME. In irradiated tumor cells, DNA-PK inhibitors did not induce type I IFN signaling through the classical cGAS/STING signaling pathway, but in a manner dependent on RNA polymerase III (POL III), retinoic acid-inducible gene I (RIG-I), and antiviral signaling protein (MAVS), while also promoting the expression of PD-L1, reversing the poor immunogenicity of tumor cells and increasing sensitivity to anti-PD-L1 antibodies (Wang et al., 2022). DNA-PK inhibitors accelerate the formation of micronuclei in the nucleus, a key factor in the generation of cytoplasmic DNA. The generation of DNA activates the cGAS/STING signaling pathway, enhancing the efficacy of immunotherapy. The STING signaling pathway, which increased PD-L1 expression in irradiated tumor cells, further enhanced the efficacy of ICB(151). Meanwhile, DNA-PK inhibitors induced more tumor cells to enter mitosis, resulting in their death. This is partly attributed to the excessive activation of the ATM/P53 signaling axis by DNA-PK inhibitors, which prolongs cell cycle arrest and induces premature apoptosis (Carr et al., 2022). Most UCs, such as prostate, bladder, and renal cell carcinomas, are P53-deficient, and DNA-PK inhibitors protect normal cells with P53-dependent proliferation from mitotic death (Leszczynska et al., 2015; Jung et al., 2021; He et al., 2022b; Chaudagar et al., 2023). For P53-deficient tumor cells, DNA-PK inhibitors activate the ATM/P53 signaling axis and thus induce strong cell-killing activity (Carr et al., 2022). In addition, a preclinical study confirmed the therapeutic efficacy of DNA-PK inhibitors in ATM-deficient tumors, probably because ATM and DNA-PK are jointly involved in the NHEJ process and the survival of ATM-deficient tumor cells is highly dependent on DNA-PK signaling, which activates the DNA damage repair process (Xue et al., 2022). The results of a series of preclinical studies suggest that P53 status and ATM deficiency may be predictive biomarkers for the sensitivity of patients to DNA-PK inhibitors.

#### 3.9.3 Clinical trials of combination therapy with DNA-PK inhibitors and ICIs in UC

Two DNA-PK inhibitors (M3814 and CC-115) are currently in phase I/II clinical trials. NCT01353625 is a phase I clinical trial of CC-115 in prostate cancer with clinical endpoints of DLT and maximum observed blood concentration (Cmax). The results showed that after treatment with CC-115, 64% of patients with prostate cancer achieved disease stabilization. The most common AEs were gastrointestinal, skin, and subcutaneous tissue reactions, including diarrhea, nausea, rash, and maculopapular rash. However, the overall adverse effects were mild, and most patients tolerated CC-115 well and achieved good efficacy, making it a promising new anti-cancer treatment (Munster et al., 2019). NCT04071236 is a phase I/II clinical trial designed to investigate the optimal dose and efficacy of M3814 in combination with avelumab in prostate cancer, with the clinical endpoints of DLT and radiologic PFS (rPFS). The trial is currently in the recruitment phase. NCT03724890 is a completed Phase I clinical trial combining M3814 and avelumab in solid tumors, with DLT and Cmax as clinical endpoints, to evaluate the safety and pharmacokinetics of this combination therapy. Although the results of this trial have not yet been published, they may provide a new basis for the combination of DNA-PK inhibitors and ICIs in UC and solid tumors (Table 2).

## 4 Conclusion and outlook

**FIGURE 3:**
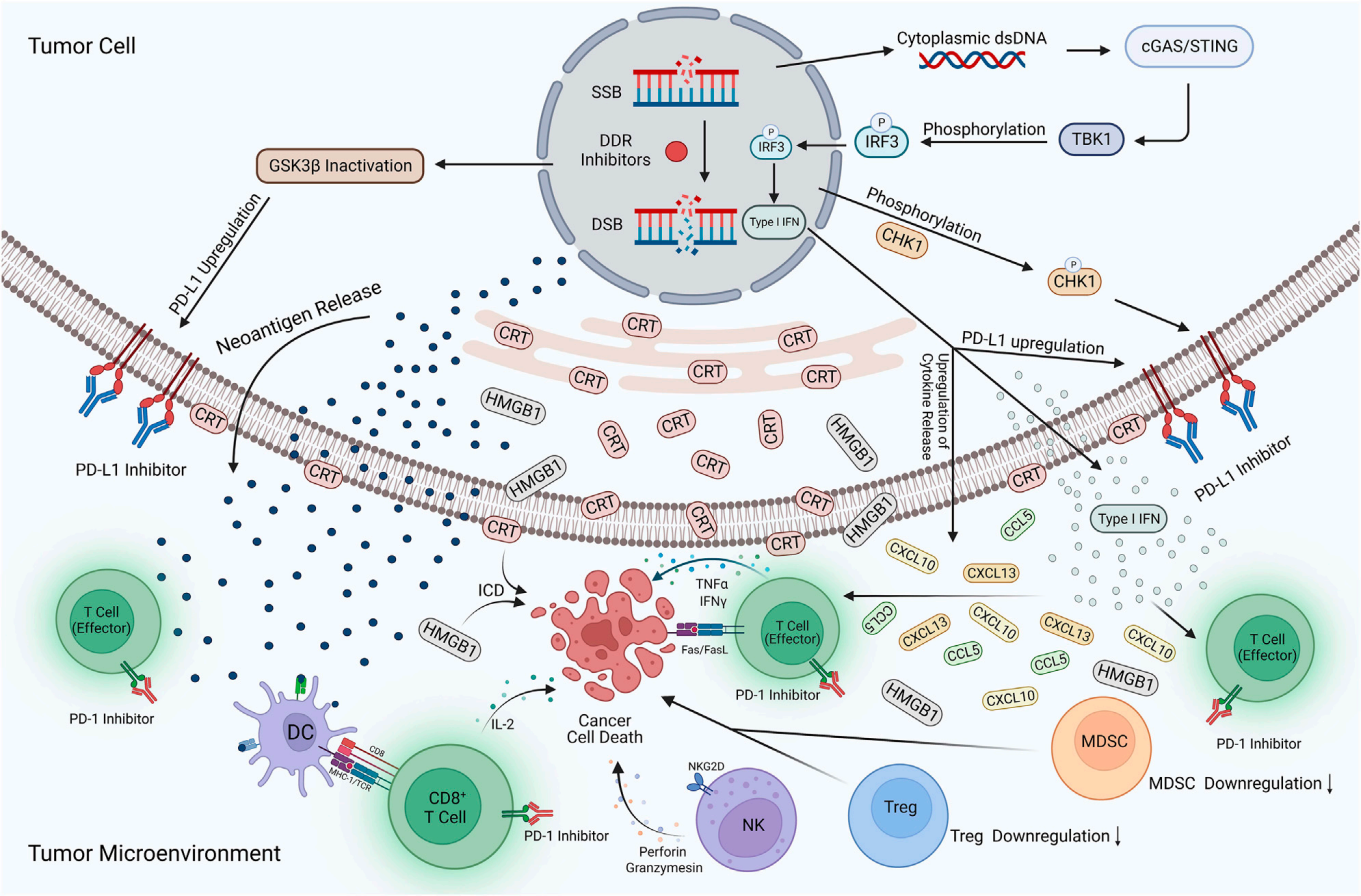
Potential mechanisms of DDR inhibitors in combination with ICIs for UC. The action of DDR inhibitors on tumor cells leads to elevated TMB expression, prompting more expression of neoantigens and their release into the TME. In addition, cytoplasmic dsDNA is released into the cytoplasm to activate the cGAS/STING/TBK1/IRF3 signaling pathway. Activating this pathway contributes to the development of type I IFN responses, while many pro-inflammatory cytokines such as type I IFN, CXCL10, CCL5, and CXCL13 are produced and enter the TME. The development of type I interferon response, inactivation of GSK3β, and phosphorylation of the CHK1 protein also increased PD-L1 expression on the tumor surface. DDR inhibitors reversed the immunosuppressive TME, with a large amount of CRT translocation exposed to the tumor cell surface and HMGB1 release into the TME, contributing to the development of ICD. In addition, the expression of MDSCs and Tregs is significantly reduced, DC cells activate effector T-cells upon uptake of neoantigens, and large amounts of cytokines are released, including IL-2, IFNγ, and TNF-α. NK cells also release perforin and granzymes, which have a killing effect on tumor cells. ICIs occupy PD-L1 targets on the surface of tumor cells and PD-1 targets on T-cells, avoiding the binding of PD-L1 to PD-1 and reducing the immune escape response of tumor cells. Applying this combination therapy led to a substantial enhancement of natural and adaptive immunity and an increase in the body’s immune surveillance of tumor cells, improving the effectiveness of immunotherapy. Created with BioRender.com.

With increasing research, the vital role played by DDR and immunomodulation in the development of tumorigenesis is increasingly recognized. Therefore, combining DDR inhibitors with ICIs in UC is a novel and highly promising therapeutic strategy. This combination therapy is acknowledged for the following reasons (Figure 3). First, DDR inhibitors enhanced the immunogenicity of tumor cells. DDR inhibitors leads to the loss of DNA fidelity, resulting in genomic instability, which partially leads to an increase in TMB. Tumor cells with high TMB expression tend to produce more neoantigens, increasing the immunogenicity of tumor cells and making them more sensitive to immunotherapy (Joshi et al., 2020). Second, DDR inhibitors activate immune-related signaling pathways. DDR inhibitors accelerate the production of micronuclei in the nucleus, which is an important factor in dsDNA production. The production of dsDNA activates the cGAS-STING-TBK1-IRF3 signaling pathway and promotes the release of type I IFN (Chiu et al., 2023). Meanwhile, DDR-related inhibitors promote the SUMO-ization of SHP1 at lysine 127, inhibiting the TRAF6-STING-p65 signaling pathway and activating the nonclassical STING signaling pathway. The activation of both STING signaling pathways promotes type I IFN expression, enhances the natural and adaptive immune function of the body, and increases immune surveillance of tumor cells by the body which enhances the effect of immunotherapy (Liu et al., 2023). The third is the regulation of the expression of PD-L1. Type I IFN can bind to IFN receptors on macrophages and activate downstream JAK1 and STAT signaling, increasing PD-L1 expression (Gao et al., 2023). In addition, DDR inhibitors prevented ubiquitination and degradation of PD-L1 by activating the STING signaling pathway, leading to GSK3β inactivation, while promoting the binding of miR-513 to PD-L1 and phosphorylation of CHK1 protein; this enhanced the stability of PD-L1 and promoted the expression of PD-L1 (Jiao et al., 2017; Xue et al., 2020; Sun et al., 2022; Zhang et al., 2022). In addition to promoting PD-L1 expression, some DDR inhibitors reduce its expression level by inhibiting the ATR-CHK1 signaling pathway, activating the CDK1-SPOP axis, and promoting the ubiquitination and degradation of PD-L1 (Tang et al., 2021). The regulation of PD-L1 expression by DDR inhibitors may activate different anti-tumor mechanisms in the body. However, the anti-tumor immune response is undeniably enhanced, and the efficacy of immunotherapy is improved. Finally, the immunosuppressive TME was reprogrammed. DDR inhibitors promoted the increase of ISG expression (including CCL5 and CXCL10) and the release of other inflammatory cytokines by activating the STING signaling pathway (Yu et al., 2022). The release of inflammatory cytokines can recruit and activate immune-related cells in the TME, enhancing the body’s ability to kill tumor cells and induce immunogenic cell death (Xie et al., 2022). Simultaneously, DDR inhibitors reduce the expression of the immunosuppressive MDSC population, reverse the immunosuppressive TME, and significantly improve the efficacy of immunotherapy (Sen et al., 2019b).

Based on promising data obtained in preclinical trials on malignant tumors, clinical trials have been conducted at many institutions for the combined treatment with DDR inhibitors and ICIs in UC, including prostate, uroepithelial, and renal tumors. Despite their achievements, these preclinical trials still face many challenges and problems.

First, indications and treatment regimens for combination therapy are poorly established. Different DDR inhibitors and ICIs may have different synergistic effects for different types or stages of UC, and different doses may affect the safety and efficacy of the combination therapy. The selection of the appropriate patient population is also critical, and more clinical trials are needed to determine which patients will benefit more from combination therapy. Second, combining DDR inhibitors with ICIs may increase the risk of toxicity and side effects, such as immune-related adverse events, affecting patients’ quality of life and treatment adherence, so means of reducing the toxic effects of combination therapy need to be addressed immediately (Shi et al., 2022). The mechanism of combination therapy requires continued investigation, and more effective biomarkers or prognostic indicators are needed to detect patients’ responses to combination therapy and predict the risk of adverse reactions. Timely intervention and management of patients who experience adverse reactions are also needed to maximize the therapeutic effect and minimize the harm caused by toxic side effects. Finally, the occurrence of drug resistance or treatment failure in patients must still be considered, and research and development of new treatment strategies are needed to further enhance the efficacy of combination therapies by using other drugs or treatments in combination.

Developing combination therapies requires strengthening international collaboration and sharing research results and data. More funding and support are recommended to promote research and development of this therapeutic strategy to serve clinical needs better. In conclusion, combining DDR inhibitors and ICIs in UC is a promising therapeutic strategy. Although many challenges and problems remain, with the development of science and technology and in-depth investigation, this combination therapy is expected to provide more options, hope for UC patients and improve their quality of life and health status.

## Abbreviations

UC Urinary cancer; ICB immune checkpoint blockade; ICIs Immune checkpoint inhibitors; DDR DNA damage repair; SSB DNA single strand damage; DSB DNA double-strand damage; MSI Microsatellite Instability; TMB Tumor mutation burden; MMR Mismatch repair; BER base excision repair; NER nucleotide excision repair; HRRR homologous recombination repair; NHEJ non-homologous end-joining repair; ssDNA Single-stranded DNA; PARP poly ADP ribose polymerase; TME Tumor Microenvironment; CRT Calreticulin; ATM ataxia-telangiectasia mutated kinase; EMT Epithelial-mesenchymal transition; ATR ataxia-telangiectasia and Rad3-related protein; dsDNA double-stranded DNA; SCLC Small Cell Lung Cancer; DNA-PK DNA-dependent protein kinase; MDSC Myeloid suppressor cells; CHK1/2 Checkpoint kinase 1/2; CDK4/6 Cell cycle-dependent kinase 4/6; RB Retinoblastoma protein; DAMPs Damage associated molecular patterns; ERV endogenous retrovirus; ICD Immunogenic cell death; ISG IFN- stimulated gene; SUMO small ubiquitin-like modifier.
